# Some socially poor but also some socially rich adolescents feel closer to their friends after using social media

**DOI:** 10.1038/s41598-021-99034-0

**Published:** 2021-10-27

**Authors:** J. Loes Pouwels, Patti M. Valkenburg, Ine Beyens, Irene I. van Driel, Loes Keijsers

**Affiliations:** 1 Amsterdam School of Communication Research, University of Amsterdam, Amsterdam, The Netherlands; 2 Behavioural Science Institute, Radboud University, Nijmegen, The Netherlands; 3 Department of Psychology, Education & Child Studies, Erasmus University, Rotterdam, The Netherlands

**Keywords:** Psychology, Human behaviour

## Abstract

Who benefits most from using social media is an important societal question that is centered around two opposing hypotheses: the rich-get-richer versus the poor-get-richer hypothesis. This study investigated the assumption that both hypotheses may be true, but only for some socially rich and some socially poor adolescents and across different time intervals. We employed a state-of-the-art measurement burst design, consisting of a three-week experience sampling study and seven biweekly follow-up surveys. Person-specific analyses of more than 70,000 observations from 383 adolescents revealed that 12% of the socially rich adolescents (high in friendship support or low in loneliness) felt closer to their friends after using social media, as opposed to about 25% of the socially poor adolescents (low in friendship support or high in loneliness). However, only 1 to 6% of all adolescents (socially rich and poor) felt closer both in the short- and longer-term. These results indicate that the rich-get-richer and the poor-get-richer hypotheses can hold both, but for different adolescents.

## Introduction

Now that today’s adolescents can be constantly connected with friends via social media, a relevant question is whether social media use can help them obtain social capital, the links and bonds formed through friendships and acquaintances^1^. Much of the social media debate centers around the question who benefits from using social media in terms of social capital, and who does not. Two opposing hypotheses prevail in the literature: The rich-get-richer and poor-get-richer hypotheses. The present study addresses these hypotheses with regard to friendship closeness, because developing and maintaining close (i.e., supportive, accessible, responsive, and intimate^2^) friendships is one of the most significant components of social capital in adolescence^3^. The rich-get-richer hypothesis poses that especially socially rich adolescents (high on friendship support or low on loneliness) may benefit from social media use, because their social media use reinforces and strengthens their already existing positive relationships with friends^4,5^. Conversely, the poor-get-richer hypothesis proposes that especially socially poor adolescents (low on friendship support or high on loneliness) benefit from using social media, because social media use may help these adolescents compensate for the loneliness and lack of friendship support in their daily lives^6,7^.

Studies that examined the rich-get-richer and poor-get-richer hypotheses with regard to friendship closeness have yielded inconsistent results^3,5,7,8^. Some studies found that mainly socially rich adolescents benefit from social media use^3^, others that mainly socially poor adolescents benefit from social media use^8^, and yet others supported both hypotheses^7^. An important explanation for this inconsistency in the literature may be that previous studies have overlooked three theoretical principles of human development^9–11^ and media effects^12,13^. These principles state that (1) media effects are intra-individual changes in thoughts, emotions, or behaviors that occur within persons as a result of their media use, (2) short-term media effects (e.g., across hours or days) may differ in strength and sign from longer-term effects (e.g., across weeks, months, or years), and (3) short- and longer-term media effects may differ from person to person^9,10,12,14,15^.

The aim of the current preregistered study was to apply the three principles of media effects to investigate the rich-get-richer and poor-get-richer hypotheses. To meet this aim, we used data from a larger project on adolescents’ social media use and psychosocial functioning, which employed a state-of-the-art measurement burst design^16^ that combined closely spaced Experience Sampling Methodology (ESM) assessments with biweekly longitudinal surveys. In the present study, we analyzed more than 70,000 observations from 383 8th and 9th graders to investigate the short-term within-person effects of social media use on momentary friendship closeness among socially rich and socially poor adolescents (Principle 1). Moreover, we examined how these short-term effects accumulated in longer-term changes in friendship closeness across three months (Principle 2). In addition, using an *N* = 1 approach, we also explored person-to-person heterogeneity in these short- and longer-term effects (Principle 3).

### Principle 1: Media Effects Occur Within Persons

The first principle of media effects is that media effects are within-person changes in cognitions, emotions, and behavior due to media use^12^. However, although most media scholars would agree on this principle, studies on the associations of social media use with friendship closeness often confound such within-person changes with variance due to between-person differences^3,7^. This confound is now increasingly being recognized as problematic, as within-person and between-person associations often differ from each other^17^, and could even be opposite in sign. For instance, recent work of Pouwels et al. has shown that social media use was *positively* related to friendship closeness at the between-person level, but *negatively* at the within-person level^18^*.* These findings suggest that even though adolescents with higher average levels of friendship closeness than their peers may use social media more frequently than their peers, this between-person finding does not preclude that adolescents with relatively high average levels of closeness feel *less* momentary friendship closeness after having used social media.

As within-person and between-person associations may differ, drawing conclusions about within-person changes (or media effects) based on between-person associations may lead to flawed conclusions^14,19,20^. In the present study, we therefore avoid this within-/between-person confound by investigating (a) how social media use affects friendship closeness within each adolescent and (b) whether and how these within-person effects depend on adolescents’ social richness and poorness.

### Principle 2: Short-Term Media Effects May Differ From Longer-Term Media Effects

The second principle of media effects is based on theories of human development, which focus on whether, how, and why individuals change over time^10,11,15^. These theories suggest that short-term effects of media use may shape longer-term effects^15,21^, but that such short-term effects may differ in strength and sign from longer-term effects. For example, lonely adolescents may use social media to compensate for a lack of close friendships in their offline world by initiating new friendships online^6,22^. In the short-term, these adolescents may experience a positive effect of social media use on their perceived closeness to these online friends. These short-term rewards may motivate them to spend an increasing amount of time on social media, but perhaps at the expense of their already scarce offline interactions with friends^6,22^. Moreover, these online friendships may be too ephemeral to transition to their offline world^22^. Eventually, the positive short-term effects of social media use on friendship closeness among these adolescents may result in longer-term negative effects on friendship closeness^6,22,23^.

To fully understand whether socially poor or socially rich adolescents benefit from social media use we cannot rely solely on either a short-term or a longer-term study. Instead, we need to combine multiple time scales to investigate how short-term media effects drive longer-term effects on friendship closeness among adolescents^11,15^. In the present study, we presented adolescents with six ESM surveys per day to assess their social media use and friendship closeness. We used these ESM surveys to detect the short-term effects of social media use on fluctuations in friendship closeness, and how these short-term effects differ for socially poor and socially rich adolescents. Moreover, to examine how these short-term effects predicted adolescents’ longer-term change in friendship closeness, we also administered seven biweekly assessments of friendship closeness following upon the ESM study.

### Principle 3: Media Effects Differ From Person to Person

The final principle of media effects is based on theories of transactional development^9^ and the Differential Susceptibility to Media Effects Model^13^, which postulate that media effects differ from person to person, based on a unique combination of dispositional and socio-contextual characteristics. To the best of our knowledge, previous studies have only investigated the rich-get-richer and poor-get-richer hypotheses at the aggregate level (e.g., using a correlational or group-based moderation approach), resulting in one effect size for the entire population or subpopulation of socially rich and poor adolescents. However, lonely adolescents cannot be considered as a homogeneous group, for example, because they differ in their self-perceived friendship experiences^24^. According to the Differential Susceptibility to Media Effects Model and theories on transactional development, it is therefore well possible that some socially poor adolescents get richer due to their social media use, whereas others get poorer, and yet others experience no discernable changes. Likewise, while some socially rich adolescents may get richer, others may get poorer, and yet others may not experience any changes. In our study, we incorporated an *N* = 1 approach, which allowed us to examine how many socially rich adolescents behaved in a manner consistent with the rich-get-richer hypothesis and how many socially poor adolescents in a manner consistent with the poor-get-richer hypothesis.

### The present study

**Figure 1:**
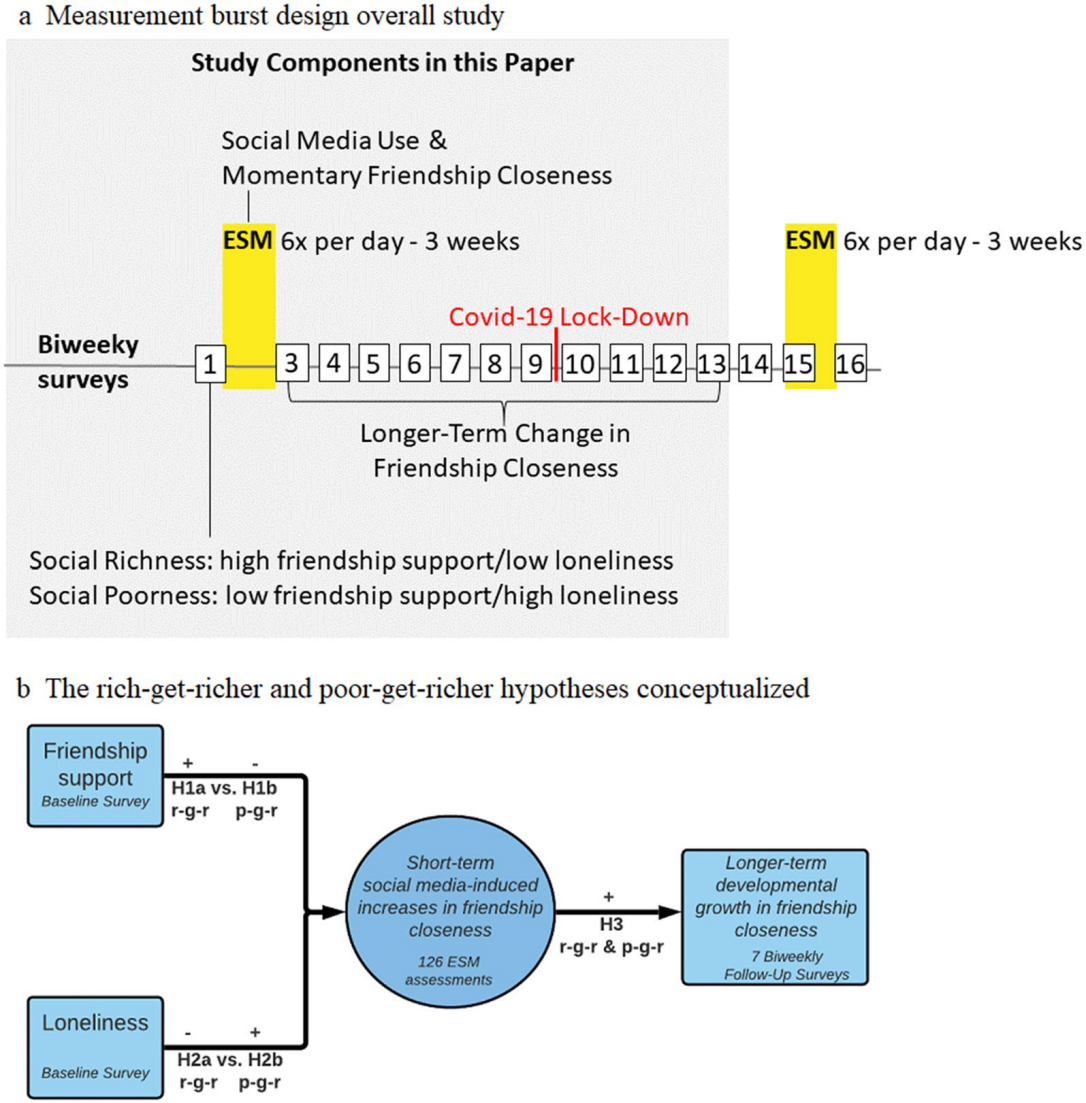
Study design (Fig. 1a) & conceptual model (Fig. 1b). *Note* (**a**)*.* The main analyses regarding the longer-term change in friendship closeness were based on biweekly survey 3 to 9 (i.e., until the Covid-19 school lock-down). The exploratory analyses also included survey 10 to 13 to examine the impact of the lock-down on the longer-term change in friendship closeness. *Note* (**b**) R-g-r is rich-get-richer hypothesis; p-g-r is poor-get-richer hypotheses. Short-term social media-induced increases in friendship closeness are operationalized as a positive person-specific within-person effect of social media use with friends on friendship closeness. Longer-term developmental growth in friendship closeness is operationalized as a positive person-specific effect of week of study on friendship closeness.

This preregistered study is part of a larger study on the psychosocial consequences of adolescents’ social media use (https://osf.io/uxnm8/). The larger study employed a measurement burst design that consisted of two three-week ESM bursts (scheduled six months apart), two baseline surveys, and 16 biweekly surveys (see timeline in Fig. 1a). The current study is, in part, a secondary data-analysis of Pouwels et al. (2021) who found substantial heterogeneity in the sign and strength of the short-term effect of social media use with friends on friendship closeness based on the first ESM burst^18^. The present study tested the rich-get-richer and poor-get-richer hypotheses while incorporating a different theoretical framework that led to three extensions. A first extension of Pouwels et al. is that we investigated whether and to what extent the short-term effects of social media use on friendship closeness depend on baseline levels of friendship support and loneliness. Friendship support is defined as companionship and affection^2^. Loneliness refers to the negative emotional response to an experienced discrepancy between actual and desired social relationships^25^.

A second extension of Pouwels et al. is that we examined whether the short-term effects of social media use on friendship closeness are related to socially rich and poor adolescents’ longer-term change in friendship closeness across seven biweekly follow-up surveys (i.e., survey 3 to 9). After biweekly survey 9, the Dutch schools had to close due to the COVID-19 lockdown. To examine the impact of COVID-19 on adolescents’ friendship closeness, we conducted some additional exploratory analyses based on biweekly survey 10 to 13. Finally, as a third extension, we examined heterogeneity in the short-term and longer-term effects within subgroups of socially rich and poor adolescents.

In line with modern theories of human development^9,10^ and media effects^12^, we proposed that the rich-get-richer hypothesis would be supported if (H1a) friendship support would positively or (H2a) loneliness would negatively predict adolescents’ short-term social media-induced increases in friendship closeness, and (H3) if these short-term increases would subsequently result in longer-term growth in friendship closeness. In contrast, the poor-get-richer hypothesis would be supported if (H1b) friendship support would negatively or (H2b) loneliness would positively predict adolescents’ short-term social media-induced increases in friendship closeness, and (H3) if these short-term increases would subsequently result in longer-term growth in friendship closeness. These hypotheses are summarized in Fig. 1b.

In addition to testing these hypotheses, we investigated whether adolescents’ longer-term growth in friendship closeness would depend on friendship support (RQ1) and loneliness (RQ2), and, if so, whether these effects would be mediated (explained) by adolescents’ short-term social media-induced increases in friendship closeness (RQ3 & RQ4). Finally, in line with the third principle of media effects and inspired by Grice et al.^26^, we assessed for how many socially *rich* adolescents the *rich-get-richer* hypothesis would be confirmed in the short- and longer-term (RQ5), and, conversely, for how many socially *poor* adolescents the *poor-get-richer* hypothesis would be confirmed in the short- and longer-term (RQ6).

## Results

### Descriptive Statistics and Correlations

On average, adolescents indicated in the baseline survey that they experienced high levels of friendship support (*M* = 4.18, range 1–5) and low levels of loneliness (*M* = 1.59, range 1–5, see Table 1). The 3-week ESM assessments (6 per day) revealed that in 41% of all ESM measurements, adolescents had used social media with friends in the previous hour. Adolescents’ person-mean levels of friendship closeness (*M* = 4.47, Range 1–7) were somewhat lower when measured via ESM than when assessed via the seven biweekly surveys following the ESM (*M* = 5.72, Range 0–7).

**Table 1:** Descriptive statistics and correlations of main study variables.

	Descriptive Statistics^a^					Correlations^b^				
Variable	*n*	No. of observations	Theoretical range	*M*	*SD*	1	2	3	4	5
1. Friendship support (Baseline)	382	382	1–5	4.18	.63	**–**				
2. Loneliness (Baseline)	383	383	1–5	1.59	.76	−.23***	–			
3. Social Media Use With Friends (ESM)	383	34,920	0–1	.41	.26	.09	−.01	–	−.05***	
4. Friendship Closeness (ESM)	383	35,043	1–7	4.47	1.29	.22***	−.21***	.11*	–	
5. Friendship Closeness (Follow-Up)	373	2,208	0–7	5.72	1.29	.25***	−.28***	.06	.41***	–

^a^Means of friendship closeness and social media use with friends represent the average of the person-mean scores. For social media use with friends, these person-mean scores reflect the proportion of occasions during which participants used social media with friends (i.e., adolescents used social media with friends at 41% of the occasions).

^b^Within-person correlations are presented above the diagonal and between-person correlations below the diagonal.

*P ≤ .05, **P ≤ .01, ***P ≤ .001.

Between-person correlations revealed that adolescents who experienced more friendship support during the baseline felt less lonely (see Table 1). Those who had more friendship support and those who were less lonely experienced more friendship closeness during the ESM and across the three-month follow-up. These findings confirm that friendship support and loneliness are unique indicators of social richness and poorness. Adolescents who experienced more friendship closeness across the ESM assessments also reported more closeness across the follow-up assessments. Finally, as reported in Pouwels et al.^18^, adolescents who more frequently used social media with friends felt *closer* to their friends than adolescents who did less so (i.e., positive between-person correlation: *r* =  + .11). However, overall, adolescents felt *less* close to their friends when they had used social media with friends in the previous hour than when they had not used social media with friends (i.e., negative within-person correlation: *r* = -.05).

### Test of the Rich-Get-Richer and Poor-Get-Richer Hypotheses

We tested the rich-get-richer and poor-get-richer hypotheses according to our preregistered analysis plan (https://osf.io/c2j5y) with a series of autoregressive lag-1(AR^1^) Dynamic Structural Equation Models (DSEM)^27^ and longitudinal multi-level growth curve models^28^. We report both the average results in the sample, as well as the percentage of socially rich and poor participants for whom the hypotheses are confirmed based on *N* = 1 analyses.

#### Short-Term Effects of Social Media Use on Friendship Closeness (Model 1)

**Figure 2:**
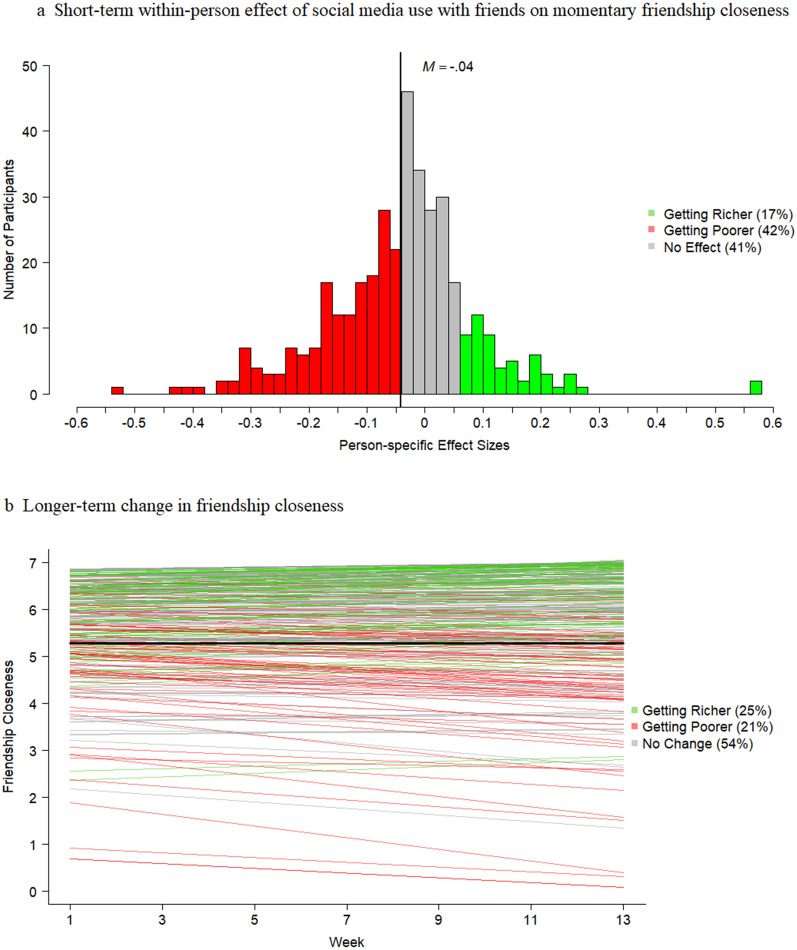
Short-term effects of social media use with friends on friendship closeness (Fig. 2a) and longer-term growth in friendship closeness (Fig. 2b).

**Figure 3:**
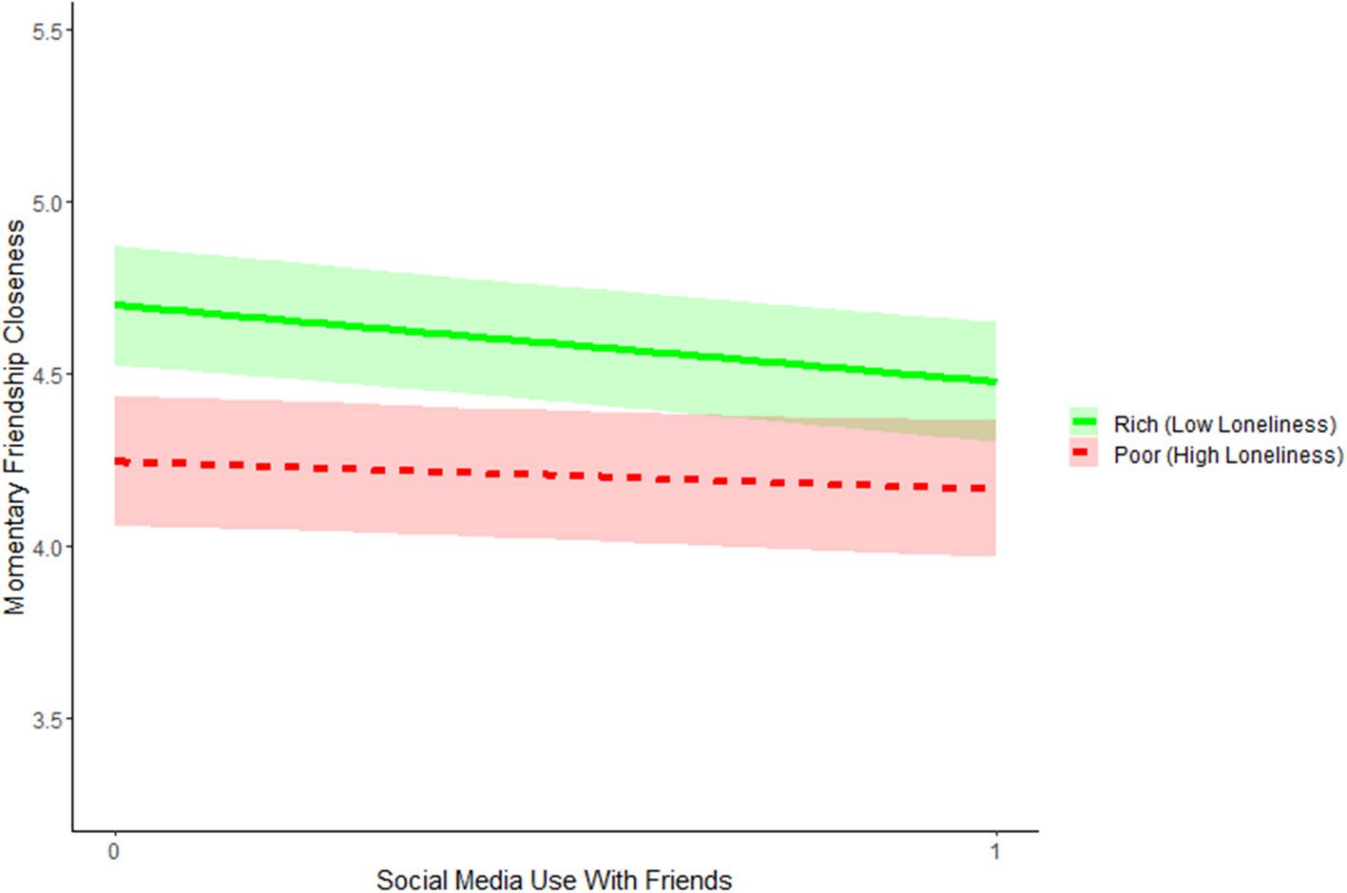
Short-term within-person effect of social media use with friends on momentary friendship closeness for adolescents with varying levels of loneliness. *Note.* The y-axis ranges from the mean + / − 1 SD. Subgroups of socially rich and poor adolescents were created based on the Mean + /1 SD, with the exception of low loneliness for which we used the absolute minimum of 1. Social media use with friends was dummy-coded (0 = no social media use with friends; 1 = social media use with friends).

As a first step, we investigated the short-term effect of social media use with friends on friendship closeness (Model 1.1, Table 2) and whether this effect would depend on friendship support and loneliness (Model 1.2, see paths H1 & H2 in Fig. 1b). In our baseline autoregressive DSEM model (Model 1.1, Table 2) we found a very small negative overall short-term within-person effect of social media use with friends on friendship closeness (β = -.041). However, there was substantial heterogeneity across adolescents in the strength and sign of this short-term effect, with person-specific effect sizes ranging from β = -.536 to β =  + .561 (see Fig. 2a). To test whether the differences in the person-specific within-person effects of social media use on friendship closeness would depend on adolescents’ social richness and poorness (H1a vs. H1b & H2a vs. H2b), we added between-person predictors to the model (i.e., friendship support and loneliness; Model 1.2). The short-term effects of social media use on friendship closeness did not differ across those with higher and those with lower levels of friendship support. However, the degree of loneliness was related to the effects of social media use on how close an adolescent feels to his/her friends (β =.139, see Model 1.2). As shown in Fig. 3, adolescents low on loneliness were more likely to experience short-term decreases in friendship closeness after using social media than adolescents who high in loneliness. Thus, overall, the findings disconfirm the rich-get-richer (H1a & H2a) and poor-get-richer hypotheses (H2a & H2b) in the short-term. Instead, these findings point at an unexpected pattern regarding loneliness: The socially rich get poorer.

**Table 2:** DSEM results of the short-term effects of social media use with friends (SMU) on friendship closeness (FCL) based on the ESM assesments.

	Model 1.1 (baseline model)				Model 1.2 (H1 & H2)			
	*b*	β	*p*	95% CI	*b*	β	*p*	95% CI
Fixed effect**s**								
*Within-Person*								
FCL_t−1_→ FCL_t_(AR_FCL)	0.263	**.263**	.000	[.249, .276]	0.263	**.263**	.000	[.250, .277]
SMU_t*_ → FCL _t_ (ST_SMI_FCL)	−0.160	**−.041**	.000	[−.053, −.029]	−0.160	−**.041**	.000	[−.053, −.029]
*Between-Person*								
SMU & FCL	0.043	**.125**	.010	[.024, .227]	0.036	**.110**	.018	[.007, .213]
LON & FS					−0.113	−**.232**	.000	[−.324, −.134]
FS → ST_SMI_FCL (H1a vs. H1b)					−0.059	−.063	.166	[−.189, .061]
LON → ST_SMI_FCL (H2a vs. H2b)					0.109	**.139**	.022	[.004, .264]
FS → FCL					0.363	**.176**	.000	[.075, .271]
LON → FCL					−0.292	−**.169**	.001	[−.268, −.068]
FS → SMU					0.037	.091	.045	[−.013, .195]
LON → SMU					0.002	.006	.459	[−.100, .109]

	Model 1.1 (baseline model)				Model 1.2 (H1 & H2)			
	σ^2^	*p*	95% CI		σ^2^	*p*	95% CI	
Variance (Random Effects)								
*Within-Person*								
FCL	1.977	.000	[1.947, 2.008]		1.977	.000	[1.947, 2.008]	
*R*^2^ FCL	0.127	.000	[0.119, 0.135]		0.127	.000	[0.120, 0.135]	
*Between-Person*								
FCL	1.730	.000	[1.498, 2.015]		1.609	.000	[1.399, 1.869]	
ST_SMI_FCL	0.354	.000	[0.277, 0.448]		0.346	.000	[0.274, 0.437]	
AR_FCL	0.034	.000	[0.028, 0.041]		0.033	.000	[0.027, 0.041]	
*R*^2^ ST_SMI_FCL					0.032	.000	[0.004, 0.086]	
*R*^2^ FCL					0.076	.000	[0.031, 0.135]	
*R*^2^ SMU					0.011	.000	[0.001, 0.040]	

Social media use (SMU) was dummy coded (i.e., 0 = no social media use with friends, 1 = social media use with friends); ST_SMI_FCL = short-term social media-induced change in friendship closeness; AR_FCL = the autoregressive effect of friendship closeness; FS = Friendship Support; LON = Loneliness; *b*s are unstandardized; βs are standardized using the STDYX Standardization in Mplus^55^; *p*-values are one-tailed Bayesian *p*-values^27^; significant fixed effects are depicted in bold.

#### Linking Short-Term Effects to Longer-Term Change in Friendship Closeness (Model 2)

In a second step, we examined adolescents’ longer-term change in friendship closeness (Model 2.1) and whether this change could be predicted by the short-term effects of social media use on friendship closeness (Model 2.2; see path H3 in Fig. 1b). As illustrated by the longitudinal multi-level growth model (Model 2.1; Table 3), on average, adolescents’ friendship closeness did not change significantly across the three months. However, Fig. 2b illustrates substantial heterogeneity in adolescents’ levels of friendship closeness (σ intercept = 1.295) and in the strength and sign of their 3-month change in friendship closeness (σ slope = 0.015). Counter to our expectations (H3; Model 2.2), adolescents’ short-term effect of social media use on friendship closeness was unrelated to their level and longer-term change in friendship closeness.

**Table 3:** Growth modelling results regarding the longer-term growth in friendship closeness (FCL) based on the follow-up assessments.

	Model 2.1 (baseline)				Model 2.2 (H3)				Model 2.3 (RQ1 & RQ2)			
	*b*	β	*p*	95% CI	*b*	β	*p*	95% CI	*b*	β	*p*	95% CI
Fixed effects												
*Growth Factors*												
FCL Intercept	5.723	5.035	.000	[4.489, 5.700]	5.726	5.033	.000	[4.489, 5.666]	5.718	5.228	.000	[4.644, 5.940]
FCL Slope	0.000	.001	.457	[−.033, .040]	0.000	.002	.468	[−.035, .038]	0.002	.002	.456	[−.036, 0.039]
*Correlations*												
Intercept & Slope	−0.009	−.068	.338	[−.321, .302]	−0.009	−.063	.348	[−.318, .298]	− 0.012	−.096	.300	[−.363, .344]
ST_SMI_FCL → Slope (H3)					0.066	.051	.269	[−.114, .209]				
FS → Slope (RQ1)									0.021	.076	.192	[−.100, .269]
LON→ Slope (RQ2)									0.002	.010	.449	[−.169, .171]
ST_SMI_FCL → Intercept					−1.080	−.087	.033	[−.173, .005]				
FS → Intercept									0.365	**.148**	.001	[.056, .242]
LON → Intercept									−0.394	−**.197**	.000	[−.284, −.104]

	Model 2.1 (baseline)				Model 2.2 (H3)				Model 2.3 (RQ1 & RQ2)			
	σ^2^	*p*	95% CI		σ^2^	*p*	95% CI		σ^2^	*p*	95% CI	
Variance (Random Effects)												
FCL Intercept	1.295	.000	[1.013, 1.615]		1.283	.000	[1.008, 1.607]		1.119	.000	[0.862, 1.425]	
FCL Slope	0.015	.000	[0.006, 0.026]		0.015	.000	[0.006, 0.026]		0.014	.000	[0.004, 0.027]	
*R*^2^ FCL Intercept					0.008	.000	[0.000, 0.030]		0.063	.000	[0.030, 0.109]	
*R*^2^ FCL Slope					0.004	.000	[0.000, 0.045]		0.015	.000	[0.001, 0.089]	

ST_SMI_FCL = short-term social media-induced change in friendship closeness (obtained from M1.1); FS = Friendship Support; LON = Loneliness; *b*s are unstandardized; βs are standardized using the STDYX Standardization in Mplus; *p*-values are one-tailed Bayesian *p*-values^27^; significant fixed effects are depicted in bold.

Third, we investigated whether adolescents’ longer-term change in friendship closeness would depend on their friendship support (RQ1) and loneliness (RQ2), and, if so, whether these effects would be mediated by adolescents’ short-term effect of social media use on friendship closeness (RQ3 & RQ4). Adolescents’ friendship support (RQ1) and loneliness (RQ2) did not predict their longer-term change in friendship closeness (Model 2.3). We therefore did not find evidence that the socially rich or poor adolescents got richer across the three months. Because socially rich and poor adolescents did not differ in their longer-term change in friendship closeness, the short-term effects of social media use on friendship closeness could not explain the effects of friendship support and loneliness on the longer-term change in friendship closeness (RQ3 & RQ4; Model 2.4; see non-significant indirect effects in supplementary Table S1).

### Test of the Rich-Get-Richer and Poor-Get-Richer Hypotheses at The *N* = 1 Level

**Figure 4:**
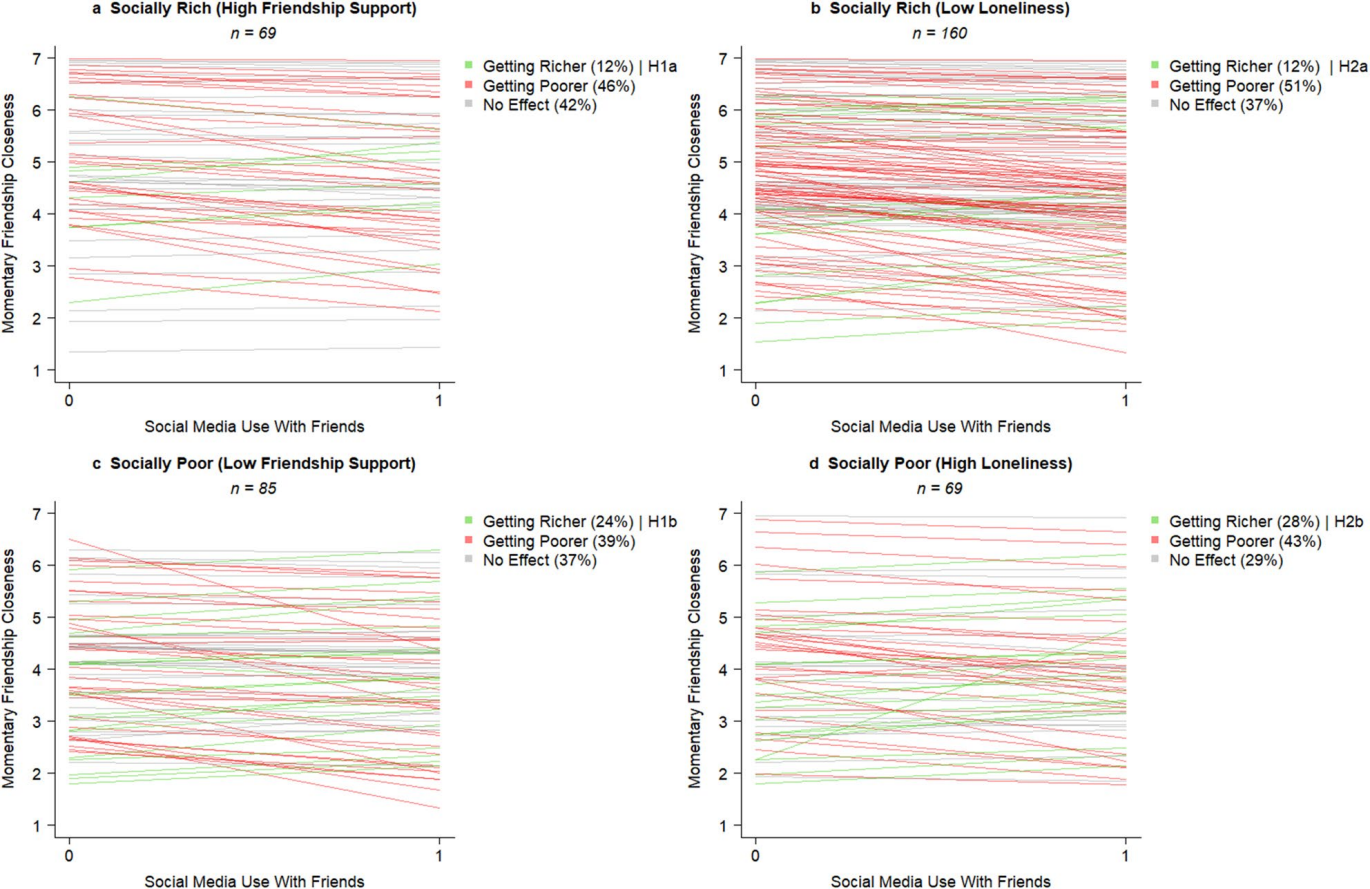
*N* = 1 Short-term effects of social media use with friends on momentary friendship closeness among socially rich (Fig. 4a & 4b) and poor adolescents (Fig. 4c & 4d). *Note.* Subgroups of socially rich and poor adolescents were created based on the Mean + /1 SD, with the exception of low loneliness for which we used the absolute minimum of 1. Social media use with friends was dummy-coded (0 = no social media use with friends; 1 = social media use with friends).

As a fourth and final step, we examined heterogeneity with regard to the rich-get-richer and poor-get-richer hypotheses. Specifically, for each of the two predictors (i.e., friendship support [RQ5] and loneliness [RQ6]), we examined how many socially rich and poor adolescents became richer or poorer in the short-term (see Fig. 4), and whether these short-term effects unfolded into longer-term increases or decreases in friendship closeness (see Table 4). In the short-term, more socially poor than rich adolescents got richer. Specifically, 24% of the adolescents with low levels of friendship support (Fig. 4c) and 28% with high levels of loneliness experienced positive short-term effects of social media use on how close they felt to their friends (Fig. 4d), as compared to 12% of the adolescents with high friendship support (Fig. 4a) or low loneliness (Fig. 4b). Thus, in the short-term, our hypotheses were only confirmed for a small group of adolescents (12 to 28%). We found that a large group of socially rich (46 to 51%) and poor (39 to 43%) adolescents got poorer instead of richer in the short-term, as indicated by a negative short-term effect of social media use on friendship closeness. Thus, in the short-term, we found stronger evidence for rich-get-poorer and poor-get-poorer effects than for the hypothesized rich-get-richer and poor-get-richer effects.

**Table 4:** Distribution of *N* = 1 effect sizes among socially rich and socially poor adolescents.

Effect size patterns		All participants		Socially rich participants		Socially poor participants	
Short-Term	Longer-Term			↑ FS *n* = 69	↓ LON *n* = 85	↓ FS *n* = 160	↑ LON *n* = 69
		*n*	%	%	%	%	%
Getting richer		**63**	**17**	**12**	**12**	**24**	**28**
	Getting Richer	15	4	1	5	4	6
	No Change	30	8	9	5	9	10
	Getting Poorer	18	5	1	2	12	12
Getting poorer		**157**	**42**	**46**	**51**	**39**	**43**
	Getting Poorer	29	8	4	10	7	7
	No Change	89	24	30	30	17	25
	Getting Richer	39	10	12	11	14	10
No effect		**153**	**41**	**42**	**37**	**37**	**29**
	No Change	82	22	22	20	20	13
	Getting Richer	40	11	12	10	10	7
	Getting Poorer	31	8	9	7	7	9
Total		**373**	**100**	**100**	**100**	**100**	**100**

↑FS = high on friendship support; ↓ LON = low on loneliness; ↓ FS = low on friendship support; ↑ LON = high on loneliness. Effects were considered as negative/positive based on the cut-off points β ≤ − .05 and β ≥ .05. Subgroups of socially rich and poor adolescents were created based on the Mean + /1 SD, with the exception of low loneliness for which we used the absolute minimum of 1.

When linking the short- and longer-term effects, the rich-get-richer and poor-get-richer hypotheses even received less support (see Table 4). Only 1 to 5% of the socially rich adolescents and 4 to 6% of the socially poor adolescents experienced both short-term social media-induced increases and longer-term increases in friendship closeness, which provides evidence for the ‘get richer assumption’ of both hypotheses. Instead, 12% of the socially poor adolescents experienced social media-induced *increases* in friendship closeness in the short-term, while their level of friendship closeness *decreased* in the longer-term. These findings support our second overarching principle of media effects which states that short-term and longer-term effects can have an opposing sign.

### Exploratory Analyses With Regard to COVID-19

To examine the impact of COVID-19 on the development of adolescents’ friendship closeness, we conducted additional exploratory analyses. Exploratory piecewise growth models (see Supplement 2 in the Supplementary Information) revealed that adolescents’ mean levels of friendship closeness showed a significant drop from *M* = 5.71 before to *M* = 4.58 after the school closure.

## Discussion

Who benefits most from using social media is an important societal question that is centered around two opposing hypotheses in the media effects literature: the rich-get-richer versus the poor-get-richer hypotheses. Previous studies that have investigated these longstanding hypotheses used a nomothetic or group-differential approach to examine whether socially rich or poor adolescents get richer^3,5,7,8^. These studies yielded inconsistent findings, which may be due to the fact that they ignored three principles of media effects that are guided by theories on development and media effects^9–13^. First, previous studies did not acknowledge that media effects occur within persons and could therefore only be examined at a within-person level. Second, short- and longer-term effects of social media may differ, and currently, there is a scarcity of insights into these developmental dynamics. Third, as media effects are heterogeneous – the rich-get-richer and poor-get-richer hypotheses may hold for only some socially rich and poor adolescents. Using a state-of-the-art measurement burst design, the present study extended previous research by testing these three principles within one and the same study, by examining the heterogeneous short- and longer-term effects of social media use upon friendship closeness.

The findings of this study supported all three principles of media effects. First, our findings confirmed that we can only truly understand the effects of social media use among socially rich and poor adolescents by investigating media effects at a within-person level. By investigating the average within-person effect across all adolescents, we found that overall, adolescents felt less close to their friends after using social media. Moreover, we found very little overall support for the rich-get-richer and poor-get-richer hypotheses, because adolescents’ loneliness and friendship support were unrelated to their short-term within-person *increases* in friendship closeness after using social media. We even found that adolescents with relatively low levels of loneliness were more likely to feel *less* close to their friends after using social media, which seems to point at a socially rich-get-poorer phenomenon. Until so far, this rich-get-poorer effect has been unnoticed in the larger body of literature on the rich-get-richer and poor-get-richer hypotheses^3,6,7^, which may be due to the fact that previous studies only examined between-person associations or did not disentangle within-person associations from between-person associations^14,20^. Future within-person studies are warranted to shed further light on this novel hypothesis that mainly the socially rich adolescents get poorer from using social media. One possible explanation is that social media use may negatively affect momentary friendship closeness among socially rich adolescents because it displaces their time spent on face-to-face interactions with their friends^29^. To fully understand the effects of social media use on momentary friendship closeness, simultaneous face-to-face interactions need to be taken into account in future research (see e.g., Achterhof et al.^30^).

Second, as predicted by theories on human development^10,15^, our findings also confirmed that short-term media effects may differ from longer-term effects. In other words, adolescents who experienced a positive or negative effect of social media on friendship closeness in the short term did not necessarily experience longer-term change in friendship closeness across the subsequent 3 months. This finding confirms the evolutionary theory of loneliness which suggests that feelings of loneliness may motivate adolescents to repair or replace perceived deficiencies in social relationships by increasing their motivation to attend to and approach social stimuli that may satisfy their need for social connection in the longer-term^31^. Based on this theory, we expect that short-term decreases in friendship closeness after using social media may lead to enhanced re-affiliation motives, which may stimulate adolescents to invest more time in their friendships, which in turn promotes their friendship closeness in the longer-term. In the end, the negative momentary within-person effects of social media use on friendship closeness may prevent a drop in feelings of friendship closeness in the long-term. This principle seems to hold for at least some adolescents, as one in four adolescents who felt less close to their friends in the short term, felt closer in the longer-term. This sensitivity to time scales may radically alter some of the existing conclusions regarding media effects. We therefore need structural and theory-guided decisions regarding the time scale on which media effects are measured^11^.

Third, as predicted by theories on transactional development^9^ and the Differential Susceptibility to Media Effects Model^13^, we found that both the short-term and longer-term effects differed from person to person. Specifically, the friendship closeness of the majority of the adolescents decreased (41%) or remained the same (41%) after using social media, whereas only 17% got richer in the short-term in terms of friendship closeness. Although we found overall very little support for the rich-get-richer and poor-get-richer hypotheses, both hypotheses were confirmed among a relatively small group of adolescents. This study, as well as other recent work^32–34^, therefore strongly stresses the need to abandon the assumption of homogeneity of media effects (which still is key- albeit implicit- in all nomothetic and between-person studies^14^). People differ, and so does their susceptibility to media effects. These differences cannot be ignored, as without a person-specific approach, the field is unable to grasp how each person is affected by his or her media use^14,35^.

Apart from providing evidence for the three principles of media effects, the implications of the study may expand the rich-get-richer and poor-get-richer hypotheses beyond the constructs of interest of this study. We investigated these hypotheses with regard to adolescents’ use of Instagram, WhatsApp, and Snapchat, and by selecting loneliness and friendship support as indicators of social richness and poorness, and friendship closeness as outcome variable. However, the hypotheses have been examined with regard to a wide range of other indicators of social richness and poorness (e.g., extraversion and self-esteem) and may also be relevant with regard to other social media platforms and messaging apps. Furthermore, by selecting friendship closeness as outcome measure, we focused on just one specific aspect of friendships. Other studies have used other indicators of friendships, such as friendship quality, intimacy, and friendship initiation^3,8,36^, as well as more general indicators of online social capital^6^. Studies that examined the rich-get-richer and poor-get-richer hypotheses in these domains used a nomothetic or group-differential approach that estimates one effect size for the entire (sub)group of socially rich or poor adolescents. These studies therefore did not acknowledge insights from developmental and media effects theories that suggest that differences within subgroups of socially rich and poor adolescents are larger than differences between these subgroups. We should therefore abandon the question *whether* socially rich or poor adolescents benefit most strongly from using social media and instead investigate *which* socially rich and poor adolescents benefit from using social media, and which adolescents do not with regard to a wide range of indicators of social capital and social media use.

Theoretically, such a research line on investigating which socially rich and poor adolescents benefit most strongly from social media could build forward on the Differential Susceptibility to Media Effects Model^13^, which states that the effects of social media differ from person to person, based on a unique combination of dispositional and socio-contextual characteristics. Even though this theory has become leading in media effects research, still very little is known about the dispositional characteristics that differentiate socially rich and poor adolescents who become richer due to their social media use from those who do not become richer, or even poorer. We are now in the unique position to answer the question what the underlying mechanisms of the short-term effects of social media use are. We therefore need to understand the differential effects of various types of social media use (e.g., private communication with friends vs. browsing through public posts), the content of social media use (e.g., humor or self-disclosure of intimate information), and the subjective interpretations of this content (e.g., feeling supported vs. misunderstood).

Finally, the present study may have important implications for practice. Practitioners and policy makers often want to know for whom social media are good or bad, to provide adolescents general advice about their media use. This study confirms that one-size advice does not fit all. Specially, we showed that some lonely adolescents may benefit from using social media, others not, and yet others may even suffer from using social media. Moreover, only a small group of the socially rich and poor adolescents benefit from using social media both in the short- and longer-term. If reality is so nuanced, it may be difficult to help adolescents based on general advice. We can only effectively help adolescents by providing them tailored, person-specific advice based on their own, unique short- and longer-term experiences and effects. We therefore need to examine how we can implement the person-specific results of this study into eHealth applications to provide adolescents and their parents insight in whether and why social media use makes them socially richer or poorer^37,38^. Such personalized feedback based on ESM data has already been successfully implemented in clinical settings and, despite the increased burden, both therapists and patients evaluate it as an insightful addition to the usual care^39^.

## Method

### Sample characteristics

The sample of this study consisted of 383 adolescents (54% girls; *M*_age_ = 14.02 years, *SD* = 0.69) from different educational tracks: 43% were enrolled in lower prevocational secondary education (VMBO), 31% in intermediate general secondary education (HAVO), and 26% in academic preparatory education (VWO). As 96% of the adolescents was born in the Netherlands and self-identified as Dutch, the sample was a fairly accurate representation in terms of country of birth, because 95% of all 10- to 15-year-olds who lived in the school’s municipality was born in the Netherlands^40^. Moreover, the sample was also an accurate representation of the Dutch population of adolescents in terms of social media use^41^.

### Procedure

#### Sample recruitment and selection

The larger project has been approved by the Ethics Review Board of the Faculty of Social and Behavioral Sciences of the University of Amsterdam and all methods were performed in accordance with the relevant guidelines and regulations. A priori power analyses using Monte Carlo simulations were conducted for the larger project (see https://osf.io/ar4vm). These analyses indicated that a sample size of 300 participants with 42 assessments would be sufficient to reliably detect small within-person effect sizes in multi-level analyses with a power of 0.80 and significance level of 0.05. Taking potential attrition and compliance into account, we aimed for a sample size of 400 participants.

We invited all students in Grade 8 and 9 from a Dutch secondary school to participate in the study. Of the 745 students, 400 received informed consent from their parents or legal guardians for all the study participation. Of the 400 students with parental consent, 388 also provided informed assent themselves. The sample of the ESM study consisted of 387 participants, as one student withdrew from the study. We excluded four additional participants from the analyses as they did not use Instagram, WhatsApp or Snapchat more than once per week. Hence, the final sample of the present study consisted of a subsample of 383 adolescents who completed the baseline survey and participated in the ESM study, of whom 373 completed one or more follow-up surveys.

#### Baseline survey

At the start of the project, adolescents completed a baseline survey during a classroom session at school. This survey contained, amongst other scales, questions about friendship support and loneliness. Upon completion of the baseline survey, the researchers provided adolescents instructions about the ESM study and adolescents installed the ESM software application (Ethica Data) on their own mobile phone. Subsequently, adolescents completed an initial set of questions using the Ethica Data app to get familiar with the app and to indicate which social media platforms (i.e., Instagram/WhatsApp/Snapchat) they used more than once a week. They received a small gift for participating in the baseline session.

#### ESM surveys

During the three-week ESM study, we measured, amongst other variables, adolescents’ social media use and friendship closeness. They received six two-minute surveys per day at random time points within a fixed time interval (i.e., 126 ESM surveys per adolescent; 23 to 24 questions per survey). A detailed description of the exact notification scheme, reminders, and response windows can be found on the Open Science Framework (OSF; https://osf.io/tbdjq/). Adolescents received a financial compensation of €0.30 for each completed questionnaire. In addition, each day, we raffled off 4 times €25,- among all adolescents who completed all six surveys the previous day.

We sent a total of 48,258 ESM surveys. Due to some unforeseen technical issues, 428 surveys (0.89%) were not received by the participants. Accordingly, participants received 47,830 surveys, of which 35,099 (73%) were fully or partially completed. On average, adolescents completed 91.64 out of 126 surveys (*SD* = 23.74, *Min* = 11, *Max* = 125, *Mdn* = 96). Since 360 participants completed 50 assessments or more, the requirements (*N* ≥ 300 participants and *T* ≥ 50 to 100 assessments) to conduct *N* = 1 analyses were met^42,43^. Participants’ response rates were not associated with their baseline levels of friendship support and loneliness and with their mean levels of friendship closeness and social media use during the ESM.

#### Follow-up surveys

Following upon the ESM, every other two weeks adolescents received a link to a 5-min online follow-up Qualtrics survey, in which we measured their experiences of friendship closeness with regard to the previous week. Adolescents received a financial compensation of €1 to €2 per completed follow-up survey. In addition, we raffled off 4 times €25,- among all adolescents who completed the follow-up survey within two days. The 373 adolescents who participated in the follow-up part of the study completed a total of 2,208 follow-up assessments on friendship closeness, with an average of 5.92 (*SD* = 1.71, *Min* = 1, *Max* = 7, *Mdn* = 7) assessments per adolescent. Participants’ response rates were not associated with their baseline levels of friendship support and loneliness and with their mean levels of friendship closeness during the follow-up.

### Measures

An overview of the questionnaires could be find at the Open Science Framework (https://osf.io/4egth/).

#### Friendship support (baseline survey)

Adolescents’ baseline level of friendship support was measured with a short form of the Network of Relationship Inventory (NRI^2^). Like previous research^44^, friendship support was measured with one item on companionship (“In the past week, how pleasant was the relationship with your close friends?”) and one item on affection (“In the past week, how much did your close friends show that they care about you?”). Adolescents reported on a 5-point scale, which ranged from 1 “not at all” to 5 “completely”. We averaged the two items. Cronbach’s alpha was .58 and Pearson’s *r* was .37.

#### Loneliness (baseline survey)

In line with previous research, we measured adolescents’ baseline level of loneliness with the UCLA loneliness scale^4,5,45^. We used a shortened version of the scale that consisted of five negatively worded items (e.g., “How often in the previous week did you feel alone?”), which measured adolescents’ experiences of loneliness. Adolescents answered on a 5-point-scale, ranging from 1 “never” to 5 “very often”. Previous research showed that the shortened scale correlates strongly with the original 20-item scale and that all five items load on one factor^5,46^. We averaged the five items. Cronbach’s alpha was .78.

#### Short-term within-person predictor–social media use with close friends (ESM study)

We measured adolescents’ social media use with close friends for the three most frequently used social media platforms and messaging apps among Dutch middle adolescents: Instagram, WhatsApp, and Snapchat^41^. At each ESM assessment, adolescents were asked whether they had been in touch with their friends in the previous hour, and could select the following multiple answer options: yes via Instagram, yes via WhatsApp, yes via Snapchat, yes face-to-face, yes in a different way, or no. For each ESM assessment, we created one dummy score that indicated whether or not adolescents had used Instagram, WhatsApp or Snapchat with friends in the previous hour (i.e., 0 = no social media use with friends, 1 = social media use with friends).

#### Short-term within-person outcome–friendship closeness (ESM study)

In line with previous studies on social media use and friendship closeness^3,18^, we measured adolescents’ experiences of friendship closeness with the following ESM item: *“How close to your close friends do you feel right now?”.* Answer categories ranged from 1 “not at all” to 7 “completely”, with 4 “a little” as midpoint. The intra-class correlation was .41, indicating that 41% of the variance in momentary friendship closeness was due to differences between persons and the remaining 59% due to differences within persons and error.

#### Longer-term outcome–friendship closeness (follow-up surveys)

We measured adolescents’ experiences of friendship closeness in each of the follow-up surveys with one item (“How close to your close friends did you feel in the past week?”)*.* Answer categories ranged from 0 “not at all” to 7 “completely.” The intra-class correlation was 0.50.

### Statistical analyses

Unless indicated otherwise, we exactly followed our preregistered analysis plan (https://osf.io/c2j5y). We examined the rich-get-richer and poor-get-richer hypotheses by estimating two series of models in Mplus 8.5: Dynamic Structural Equation Models (DSEM)^27^ and longitudinal multi-level growth curve models^28^. We estimated the models with Bayesian Markov Chain Monte Carlo (MCMC) estimation with a minimum number of 5,000 iterations. By default, DSEM in Mplus uses uninformative priors. Before estimating the DSEM models, we checked the assumption of stationarity according to our preregistered analysis plan. The data met the stationarity assumption, as only 0.3% of the variance in friendship closeness was explained by day of the study.

In line with McNeish and Hamaker^27^, we estimated each model in different steps, which enabled us to compare the proportion of explained variance. A detailed description of the models is provided below. All models converged successfully as the Potential Scale Reduction (PSR) values were very close to 1, none of the parameter trace plots contained trends, spikes, or other irregularities, and the model fit was constant after doubling the number of iterations^47^.

#### Short-term effects of social media use on friendship closeness (model 1)

Based on the ESM data, we first investigated short-term social media-induced changes in friendship closeness (Model 1.1) and whether these changes depend on baseline levels friendship support and loneliness (Model 1.2). We estimated two-level autoregressive lag-1 models (AR[1]models) with friendship closeness as the outcome, in which we disentangled within-person effects (level 1) from between-person associations (level 2).

At the within-person level of the baseline lag-1 model (Model 1.1), we included the autoregressive effect of friendship closeness, to control for friendship closeness at each previous assessment. In addition, we estimated the average within-person effect of social media use with friends on friendship closeness. We followed the approach of McNeish and Hamaker^27^ by predicting friendship closeness from social media use with friends measured at the same measurement occasion. Temporal precedence, a condition for causality, was already implied by the fact that we measured social media use with friends with regard to a different time span (i.e., “the past hour”) than friendship closeness (“right now”)^48^.

At the between-person level of Model 1.1, we specified between-person variance around adolescents’ average level of friendship closeness (i.e., random intercept). We also included the between-person variance (i.e., random slopes) around the average within-person effect of social media use with friends on friendship closeness and around the autoregressive effect of friendship closeness. This enabled us to determine the person-specific effect sizes for short-term social media-induced changes in friendship closeness. Finally, we included the correlation between the random intercepts and slopes to obtain more stable estimations.

In Model 1.2, we investigated the rich-get-richer (H1a vs. H2a) and poor-get-richer (H1b vs. H2b) hypotheses in the short term. Specifically, we extended Model 1.1 by predicting adolescents’ short-term social media-induced changes in friendship closeness from friendship support and loneliness. At the between-person level, we included friendship support and loneliness as time-invariant predictors of the person-specific within-person effects of social media use with friends on friendship closeness (i.e., random effects). We also included the correlation between loneliness and friendship support.

#### Linking short-term effects to longer-term change in friendship closeness (model 2)

Second, we assessed adolescents’ longer-term change in friendship closeness across three months (Model 2.1) and whether this change could be predicted by the short-term effects of social media use on friendship closeness (Model 2.2). Longitudinal multi-level growth curve models were specified based on the seven biweekly follow-up surveys. We estimated two-level models with repeated follow-up assessments of friendship closeness (level 1; within-person level) nested within adolescents (level 2; between-person level).

At the within-person level of the baseline growth model (Model 2.1), we predicted friendship closeness by week of the study (i.e., linear slope). Week of the study was recoded so that the intercept reflected adolescents’ level of friendship closeness at the first follow-up assessment and the slope reflected the average biweekly within-person change in friendship closeness across the entire three-month follow-up period. We also explored whether there was a curvilinear trend in the longer-term development of friendship closeness by adding a quadratic slope to the multi-level growth models. As the quadratic slope was not significant and only explained 2% of the within-person variance in friendship closeness, we continued our analyses by estimating linear growth models.

At the between-person level of Model 2.1, we specified the between-person variance around the level of friendship closeness (i.e., random intercept). In addition, to examine heterogeneity in the longer-term change in friendship closeness, we also specified the between-person variance around the average longer-term change in friendship closeness (i.e., random linear slope). Finally, we included the correlation between the random intercept and slope.

In Model 2.2, we investigated whether adolescents’ short-term social media induced-changes in friendship closeness accumulated into longer-term change in friendship closeness following the procedure by Keijsers et al.^49^. We extended the between-person part of the Model 2.1 by including the person-specific effect sizes of short-term social media-induced changes in friendship closeness from Model 1.1 as time-invariant predictors at the between-person level. We predicted adolescents’ level (i.e., random intercept) and longer-term change in friendship closeness (i.e., random linear slope) from their short-term social media-induced changes in friendship closeness (H3).

Third, we investigated whether adolescents’ longer-term change in friendship closeness would depend on their friendship support (RQ1) and loneliness (RQ2) (Model 2.3), and, if so, whether these effects would be mediated by adolescents’ short-term effect of social media use on friendship closeness (RQ3 & RQ4; Model 2.4). In Model 2.3, we investigated whether friendship support and loneliness were related to adolescents’ longer-term change in friendship closeness, by extending the between-person part of growth Model 2.1. We included friendship support (RQ1) and loneliness (RQ2) as time-invariant predictors of the level of friendship closeness (i.e., random intercept) and longer-term change (i.e., random linear slope) in friendship closeness. We also included the correlation between loneliness and friendship support.

We finally integrated model Models 2.2 and 2.3 in Model 2.4 to investigate whether the short-term social media-induced changes in friendship closeness drive the longer-term developmental change in friendship closeness among socially rich and poor adolescents. To investigate these multi-level mediation hypotheses, we predicted the short-term social media-induced changes in friendship closeness from friendship support and loneliness. Moreover, we predicted the level (i.e., random intercept) and longer-term change (i.e., random slope) in friendship closeness from loneliness, friendship support, and the short-term social media-induced changes in friendship closeness. To investigate whether the effects of friendship closeness and loneliness on adolescents’ longer-term developmental change in friendship closeness were explained (mediated) by the short-term social media-induced increases in friendship closeness, we also specified the indirect effects (RQ3 & RQ4).

#### Test of the Rich-Get-Richer and Poor-Get-Richer hypotheses at The *N* = 1 level

As a fourth and final step, we tested the rich-get-richer hypotheses and poor-get-richer hypotheses from a person-specific *N* = 1 approach. This approach enabled us to reveal for each of the two predictors (i.e., friendship support [RQ5] and loneliness [RQ6]), for what percentage of socially rich adolescents the rich-get-richer hypothesis was confirmed and for what percentage of socially poor adolescents the poor-get-richer hypothesis was confirmed^26^. To do so, we first determined who was socially poor and who was socially rich. Adolescents who scored at least 1 *SD* above the sample mean on friendship support were considered as socially rich (*n* = 69, see Table 4). For loneliness, we considered all adolescents with the absolute minimum of 1 as socially rich (*n* = 85), as a value of minus one standard deviation below the mean was out of range. Conversely, all adolescents who scored at least 1 *SD* below the sample mean on friendship support (*n* = 160) or at least 1 *SD* above the sample mean on loneliness (*n* = 69) were considered as socially poor.

Second, we determined for what percentage of socially rich and socially poor adolescents short-term social media-induced increases in friendship closeness and longer-term increases in friendship closeness were found. Based on our previous work^50,51^, a recent meta-review of the media effects literature^52^, and recommendations on the use of effect sizes in longitudinal autoregressive studies^53^, we selected an effect size of β = .05 as the smallest effect of interest. We preregistered that an adolescent got socially richer if (a) the standardized person-specific short-term within-person effect of social media use on friendship closeness was greater than β = .05 (i.e., short-term social media-induced increase in friendship closeness; Model 1.1) and (b) the standardized person-specific slope was greater than β = .05 (i.e., longer-term growth in friendship closeness; Model 2.1).

#### Sensitivity analyses

Finally, as preregistered, we conducted sensitivity analyses to shed light on the robustness of the findings against potentially untrustworthy answer patterns (see Supplement 3 in the Supplementary Information for more details). Following the procedure of Pouwels et al.^18^, participants’ answer patterns were considered as potentially untrustworthy if at least two out of the following three criteria were violated: (1) consistent within-person response patterns, (2) no outliers, (3) absence of unserious responses to open comments (e.g., gross comments or jokes). All findings were replicated after excluding eight participants whose answers we considered as potentially untrustworthy.

## Supplementary Information

Supplementary Information.

## Data Availability

The data set generated and analysed during the current study is openly available on Figshare^54^. The preregistration of the design, sampling and analysis plan (https://osf.io/hxf7t), the questionnaires, and analysis scripts used to analyse the data for this paper (https://osf.io/9ry7j/) are available online on the Open Science Framework.
