# Supplementary material for: Some socially poor but also some socially rich adolescents feel closer to their friends after using social media
Source: Sci Rep. 2021 Oct 27;11:21176. doi: 10.1038/s41598-021-99034-0 (PMC8551228; doi:10.1038/s41598-021-99034-0)
Supplement: Supplementary file 1 — Supplementary Information. [file 41598_2021_99034_MOESM1_ESM.pdf]

Supplemental Material Belonging to

Pouwels, J. L., Valkenburg, P. M., Beyens, I., van Driel, I. I., & Keijsers, L. (2021). Some socially poor but also some socially rich adolescents feel closer to their friends after using social media. *Scientific Reports.* doi:10.1038/s41598-021-99034-0

Supplement 1

Table S1

Growth Modelling Results Regarding the Longer-Term Growth in Friendship Closeness (FCL) Based on Follow-Up Assessments and Including Indirect Longer-Term Effects.

	Model 2.4 (RQ3 & RQ4)			
	b	β	*p*	95% CI
**Fixed Effects**				
Growth Factors				
FCL Intercept	5.722	5.226	.000	[4.637, 5.888]
Correlations				
Intercept & Slope	-0.011	-.086	.314	[-.343, .295]
FS → ST_SMI_FCL (a)	-0.016	-.054	.069	[-.126, .017]
LON → ST_SMI_FCL (b)	0.013	.055	.072	[-.019, .127]
ST_SMI_FCL → Slope (H3, c)	0.077	.084	.226	[-.143, .303]
FS → Slope (RQ1)	0.022	.080	.161	[-.085, .263]
LON → Slope (RQ2)	0.002	.010	.445	[-.148, .176]
ST_SMI_FCL → Intercept (d)	-0.658	-.077	.121	[-.204, .050]
FS → Intercept	0.356	**.145**	.001	[.051, .235]
LON → Intercept	-0.387	**-.190**	.000	[-.281, -.099]
Indirect effects				
FS → ST_SMI_FCL → Slope (a*c)	-0.001		.264	[-.006, .002]
LON → ST_SMI_FCL → Slope (b*c)	0.001		.260	[-.002, .005]
FS → ST_SMI_FCL → Intercept (a*d)	0.008		.171	[-.009, .040]
LON → ST_SMI_FCL → Intercept (b*d)	-0.007		.174	[-.035, .007]

	Model 2.4 (RQ3 & RQ4)		
	σ^2	*p*	95% CI
**Variance (Random Effects)**			
FCL Intercept	1.110	.000	[0.866, 1.412]
FCL Slope	0.015	.000	[0.006, 0.026]
SMI_FCL	0.016	.000	[0.014, 0.019]
R^2 FCL Intercept	0.073	.000	[0.034, 0.126]
R^2 FCL Slope	0.031	.000	[0.003, 0.131]
R^2 ST_SMI_FCL	0.008	.000	[0.001, 0.022]

Note. ST_SMI_FCL = short-term social media-induced change in friendship closeness (obtained from M1.1); FS = friendship support; LON = loneliness; *b*s are unstandardized; βs are standardized using the STDYX Standardization in Mplus; *p*-values are one-tailed Bayesian *p*-values[27]; significant fixed effects are depicted in bold.

Supplement 2
Piecewise Growth Models

To explore how adolescents' friendship closeness developed during the COVID-19 school lock-down we estimated a piecewise growth model (Piecewise growth model M1.1) according to the procedure by Bülow et al[56]. We estimated two growth curves. The intercept of the first growth curve (i1) represented adolescents' mean level of friendship closeness directly before the COVID-19 school lockdown, and the slope (s1) reflected the normative longer-term change in friendship closeness. The intercept of the second curve (i2) could be interpreted as the mean level difference in friendship closeness directly before and directly after the start of school lock down (i.e., disequilibrium). The second slope (s2) represented the gradual changes in friendship closeness during the lock-down above and beyond adolescents' normative changes.

The models revealed that there was a significant sudden mean level change in friendship closeness after the lockdown (see Table S2). Specifically, mean levels of friendship closeness dropped with more than 1 point from $M = 5.706$ to $M = 4.583$. There were no significant normative changes in friendship closeness and friendship closeness also did not decrease significantly during the weeks of the lock-down. Thus, on average, adolescents' did not recover from the lock-down-induced decrease in friendship closeness. However, there was significant heterogeneity in adolescents' mean levels of friendship closeness and before and after the lockdown. Likewise, the normative longer-term change and change in friendship closeness during the lock-down also differed from adolescent to adolescent.

In all models, we included the correlation between the intercepts and slopes to investigate how the initial levels and change in friendship closeness before and after the school lock-down were related. Adolescents with the lowest levels of friendship closeness before the lock-down experienced the highest normative decrease in friendship closeness.

Finally, we investigated whether the disequilibrium and longer-term change in friendship closeness during the lockdown could be predicted by the main predictors of our study: adolescents' social media-induced friendship closeness (Piecewise growth model 2.2), friendship support, and loneliness (Piecewise growth model 2.3). Social media-induced friendship closeness, loneliness and friendship closeness were neither related to disequilibrium in friendship closeness (i2), nor to change in friendship closeness during the lock-down (s2).

Table S2

Main Outcomes Piecewise Growth Models

	Fit indices			Variance			Correlations		
	b	*SE*	*p*	σ^2	*SE*	*p*	*SMI_FCL*	*FS*	*LON*
Mean level FCL before COVID-19 lockdown (i1)	5.706	.081	<.001	1.753	.271	<.001	-.073	$.231^{***}$	$-.225^{***}$
Disequilibrium in FCL due to Lockdown (i2)	-1.123	.117	<.001	2.136	.425	<.001	-.011	-.087	.054
Normative Change in FCL before lockdown (s1)	-0.013	.014	.336	0.021	.007	.004	.056	.103	-.012
Change in FCL during lockdown (s2)	0.075	.045	.096	0.229	.080	.004	-.002	.059	-.002

Note. FCL = Friendship Closeness. CFI = .95; TLI = .949; RMSEA = .044. Fit indices and variances were based on the unstandardized estimates. Correlations were based on the standardized estimates based on the stdyx procedure in Mplus 8.5.

Supplement 3
Sensitivity Analysis

A preregistered sensitivity analysis was conducted to examine the robustness of our results against potential untrustworthy answer patterns. Following the procedure of Pouwels et al[18], participants' answer patterns were considered as potentially untrustworthy if at least two out of the following three criteria were violated: (1) consistent within-person response patterns, (2) no outliers, (3) absence of unserious responses to open comments (e.g., gross comments or jokes). We considered the answers of eight participants as potentially untrustworthy (see Pouwels et al.).

We reconducted our final analysis without these eight participants. A comparison of the findings of our main analyses and sensitivity analysis is presented in Table S3. As all effect sizes were relatively similar and their interpretation did not change, we could conclude that our findings were not affected by potentially untrustworthy answer patterns.

Table S3
Main Results of Analyses Including All Participants Versus Analyses Excluding Participants With Potentially Untrustworthy Answer Patterns.

	**All Participants**				**Excluding Participants with Potentially Untrustworthy Answers Patterns**			
**Model 1.2**	*b*	β	*p*	95% CI	*b*	β	*p*	95% CI
Friendship Support → ST_SMI_FCL (H1a vs. H1b)	-0.059	-.063	.166	[-.189, .061]	-.047	-.049	.238	[-.177, .078]
Loneliness → ST_SMI_FCL (H2a vs. H2b)	0.109	**.139**	.022	[.004, .264]	.118	**.147**	.015	[.014, .274]
**Model 2.2**								
ST_SMI_FCL → Slope (H3)	0.066	.051	.269	[-.114, .209]	.067	.055	.254	[-.113, .227]
**Model 2.3**								
Friendship Support → Slope (RQ1)	0.021	.076	.192	[-.100, .269]	.017	.062	.238	[-.109, .263]
Loneliness → Slope (RQ2)	0.002	.010	.449	[-.169, .171]	.005	.026	.377	[-.151, .213]
